# After 3 months of medication balloon therapy, a patient who had contrast-induced encephalopathy recovered: A case report

**DOI:** 10.1097/MD.0000000000034392

**Published:** 2023-07-28

**Authors:** Kaiyuan Kong, Anyong Chen, Guoliang Yang, Ronghua Gao, Shaohui Zhang, Lixin Liu, Xueying Chen

**Affiliations:** a College of Clinical Medicine, Jining Medical University, Jining, China; b Department of Cardiology, Jining Key Laboratory for Diagnosis and Treatment of Cardiovascular Diseases, Affiliated Hospital of Jining Medical University, Jining, China; c Postdoctoral Mobile Station of Shandong University of Traditional Chinese Medicine, Jinan, China.

**Keywords:** contrast-induced encephalopathy, drug-coated balloon, prognosis, recovery time

## Abstract

Iodinated contrast agents are extensively employed in clinical settings, with allergic reactions and renal impairment being the most prevalent adverse events. Contrast-induced encephalopathy (CIE) can present with heterogeneous clinical features, making diagnosis challenging. Prior studies on CIE have primarily documented rapid recovery within several days. However, this paper describes a case of CIE in a patient whose clinical symptoms took 3 months to fully abate.

**Patient concerns:**

A female patient, aged 54 years, received drug-coated balloon therapy for stenosis in a branch of the anterior descending coronary artery. Unfortunately, the patient developed CIE, which initially manifested as visual disturbances and subsequently progressed to gastrointestinal and limb movement issues, as well as an altered mental status, all of which occurred within a 24-hour period during hospitalization.

**Diagnoses:**

The patient was diagnosed with CIE after cerebral hemorrhage, and cerebral edema was ruled out based on the history of contrast medium administration and radiographic exams.

**Interventions and outcomes:**

Dexamethasone (10 mg/d), mannitol (100 mL/d), betahistine (500 mL), trazodone (25 mg), and hydration supplementation were given to treat CIE-related symptoms. Aspirin and clopidogrel were administered for the management of the cardiovascular ailment. The neurologist prescribed neurotrophic agents, namely, cytarabine and methylcobalamin, based on the cerebral magnetic resonance imaging findings. Despite the treatment, the patient’s ocular symptoms, including blurry vision, diplopia, and impaired intraocular retraction, persisted. Furthermore, the patient’s mental state was altered, and she continued to exhibit a depressive state during her 1-month follow-up visit.

**Lessons:**

CIE is a comparatively infrequent ailment, and its prompt identification and management are of paramount importance. Although the treatments for CIE are primarily symptomatic, it is crucial to acknowledge that the symptoms may not always subside quickly within a short duration. In conjunction with pharmacotherapy, counseling should be offered to address patients’ mental health.

## 1. Introduction

Contrast-induced encephalopathy (CIE) is an abrupt, transient neurological dysfunction that arises from the intracerebral perfusion of contrast agents.^[1]^ The employment of drug-coated balloons (DCBs) as interventional treatment for coronary artery disease is rapidly expanding.^[2,3]^ Numerous registry studies have reported cerebrovascular complication rates of 0.30% to 0.40% for percutaneous coronary intervention therapy and 0.05% to 0.11% for coronary angiography.^[4]^ Several studies have indicated that patients who undergo iodine contrast injections have an incidence of CIE of nearly 0.38%.^[5]^ CIE typically follows a benign course, and many patients exhibit recovery within a few days of its onset; however, it is relatively uncommon for the recuperation to span over weeks or months.^[6]^ The present case study highlights a CIE instance in which the duration of recovery was notably protracted.

## 2. Case report

**Figure 1.**
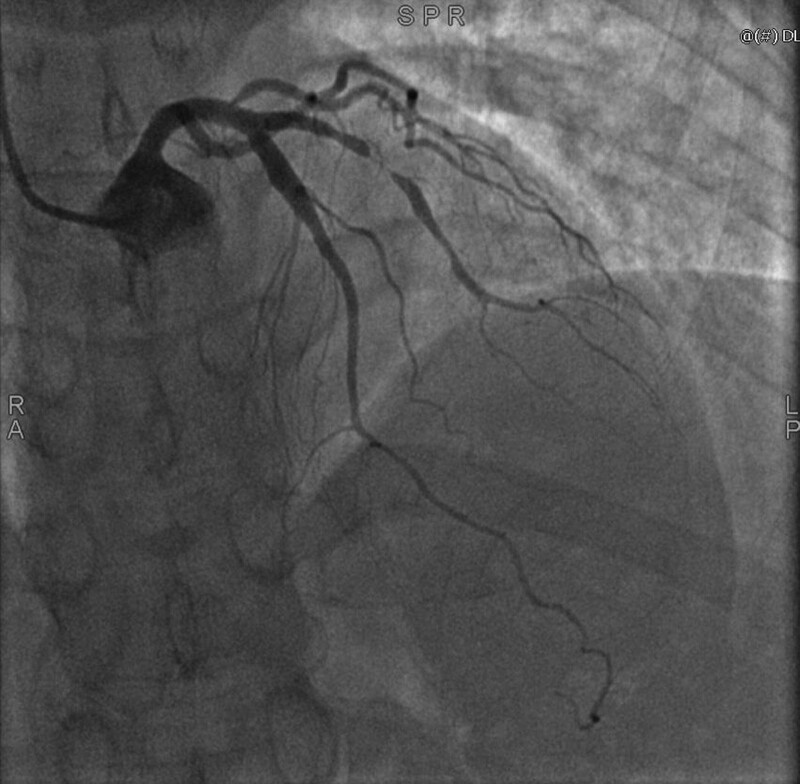
Coronary angiography findings: the most severe stenosis (90–95%) was found in the mid-D1 of mid-LAD. LAD = left anterior descending coronary.

**Figure 2.**
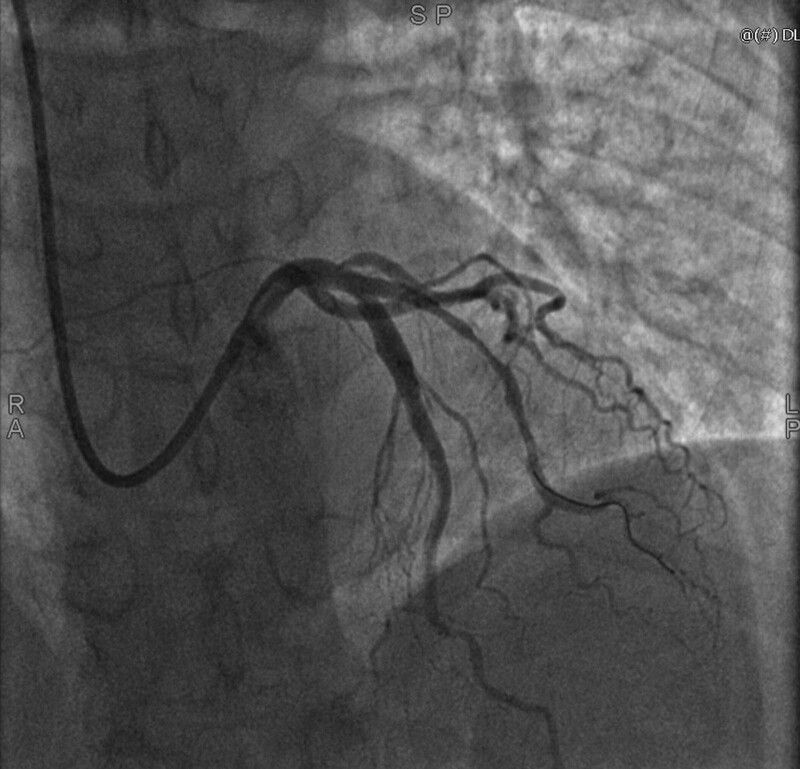
Lesion site after DCB treatment. DCB = drug-coated balloon.

The patient, a 54-year-old female, presented to the hospital with chest pain. She had no prior history of food or drug allergies, long-term medication use, hypertension, diabetes mellitus, coronary artery disease, or any other chronic ailments. The patient’s blood pressure was 134/83 mm Hg, her weight was 60 kg, her heart rate and pulse were both 75 beats per minute, and a physical examination revealed no obvious abnormalities. An electrocardiogram obtained from another hospital depicted a sinus rhythm, premature atrial beats, and ST-T alterations. The patient underwent pertinent laboratory examinations and was initially diagnosed with coronary artery disease, unstable angina, arrhythmia, and premature atrial beats. The patient’s laboratory results indicated elevated levels of triglycerides, total cholesterol, low-density lipoprotein cholesterol, and high-sensitivity troponin I (1.60 mmol/L, 5.83 mmol/L, 4.11 mmol/L, and 0.0246 ng/mL, respectively). All other tests, such as those for liver and kidney function, produced normal results. Cardiac ultrasonography demonstrated a dilated ascending aorta, a left ventricular ejection fraction of 62%, a small amount of tricuspid regurgitation, and diminished left ventricular diastolic function. The chest CT scan revealed mild emphysema, modest pleural thickening, coronary artery calcification foci, chronic inflammation, and small lung lesions. Based on the results of the coronary angiography, the patient was found to have mild stenosis (30%) in the mid-left anterior descending coronary, severe stenosis (90–95%) in the mid-D1, mild stenosis (20%) in the left circumflex artery, and moderate stenosis (40–50%) in the mid-right coronary artery, with a TIMI class III antegrade flow (as shown in Fig. 1). The patient was diagnosed with coronary artery disease affecting the D1 vessel, and the patient’s family necessitated drug balloon dilatation for her treatment. The procedure lasted approximately 1 hour and utilized approximately 200 mL of iodixanol contrast agent during the operation, as shown in Figure 2. The surgeon successfully treated the lesion with 1 drug balloon dilation using a HengYi 2.0-mm balloon dilation with 6 to 8 atm a Synephrine drug balloon 2.525 mm.

**Figure 3.**
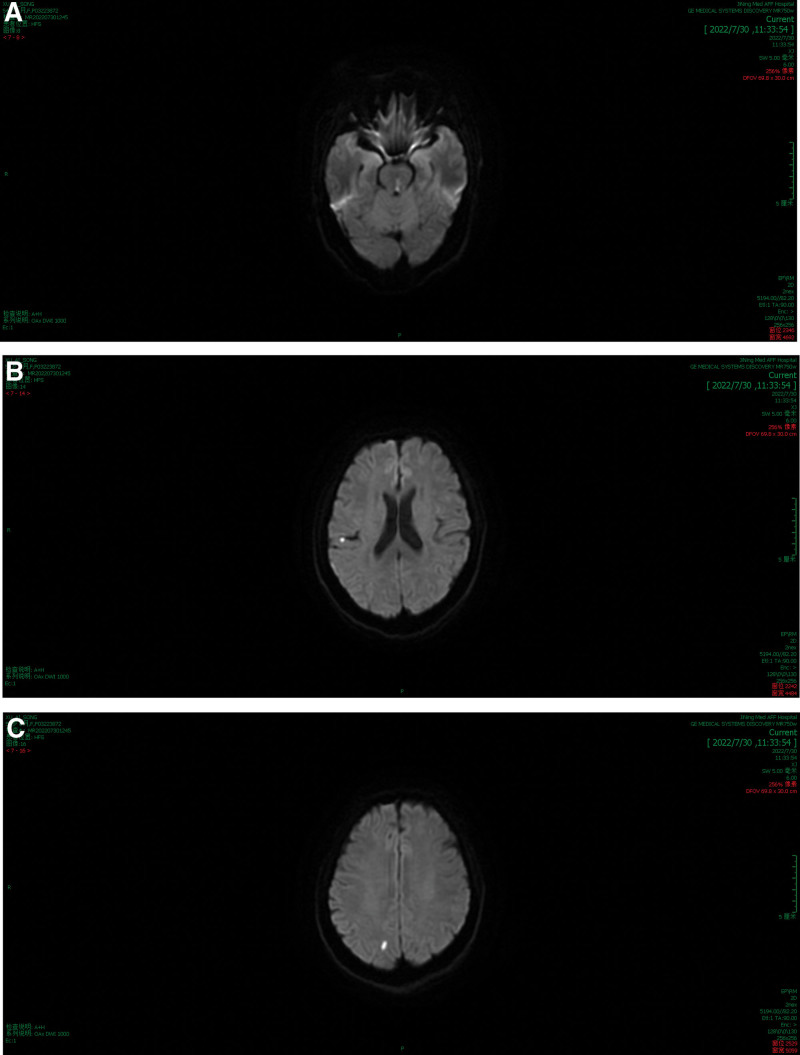
Cranial MRI showing high signal on DWI images. (A) Pons. (B) Parietal lobe. (C) Occipital lobe. DWI = diffusion-weighted imaging, MRI = magnetic resonance imaging.

Two hours after treatment, the patient experienced diplopia, blurred vision, dizziness, nausea, vomiting, mental depression, and limb motor dysfunction. However, the pupils’ reaction to light was normal. Immediately, dexamethasone (10 mg), trazodone (25 mg), betahistine (500 mg), and intravenous fluids were administered. Upon undergoing cranial magnetic resonance imaging (MRI), the patient was diagnosed with fresh infarct foci in the right parietal cortex and cerebral bridge, multiple brain foci of ischemic degeneration, and alterations compatible with mild cerebral atherosclerosis (Fig. 3). At that time, the patient’s left eye was swollen and incapable of moving internally, whereas her right eye’s movement function was entirely normal. The neurologist and ophthalmologist added prostilbene, acetylglutamate, methylcobalamin, and mannitol treatments to the patient’s regimen.

Following the treatment, the patient’s psychological condition ameliorated, allowing her to stand; however, she was still unable to ambulate, and her gastrointestinal manifestations subsided on the second day after the operation. As the days passed, the patient’s previously mentioned symptoms gradually improved, and she was ultimately discharged from the hospital. Nevertheless, at the time of discharge, the patient still presented with diplopia, blurry vision, and reduced physical vigor.

One month after the patient’s discharge, we conducted a follow-up and observed that in addition to the decreased physical strength, there was also a noticeable slowing of her thought processes. The patient appeared less responsive and communicative, exhibited reluctance toward social interactions, and displayed a pessimistic attitude. Furthermore, she continued to experience diplopia and blurred vision in her left eye, and the motor function of the eye had not yet returned to normal.

After the patient’s discharge from the hospital, it took her 3 months to fully recover. During this period, she was diagnosed with high eye pressure in her right eye at the local county hospital. It is worth noting that the patient was also struggling with psychological depression at this time. However, with constant reassurance and care from her doctor, her mood gradually improved, and she returned to her normal state by the fifth month.

## 3. Discussion

Identifying CIE in a timely manner is a formidable task due to the dearth of diagnostic criteria. CIE is an infrequent occurrence in clinical settings, and it is typically witnessed subsequent to interventions or surgeries on the cardiac and cerebral vasculature. The origin of CIE is largely ascribed to its heightened viscosity, osmolarity, and direct neurotoxicity, which can result in aberrant blood–brain barrier functionality, an imbalance in cerebrospinal fluid electrolytes, cerebrovascular spasms, and direct neural cell damage. Etiological factors, contrast agent type, dosage, surgical timing, patient age, hypertension, hyperlipidemia, hyperglycemia, and a history of cerebrovascular lesions are among the various risk factors for CIE.^[7,8]^

Based on their physicochemical structure, iodine contrast agents that are employed in clinical settings are presently categorized into the following groups: ionic monomers, for example, such as pantethine; ionic dimers, such as iodic acid; nonionic monomers, such as iodophoresol and iodofol; and nonionic dimers, such as iodixanol.^[9]^ Nonionic iodine contrast agents are preferred due to their low incidence of adverse reactions. In this particular case, the patient was administered the nonionic contrast agent iodixanol, which is deemed safer because it does not cause renal harm and has an osmotic pressure that approximates human plasma. Although the patient did not exhibit any of the aforementioned risk factors, a cerebral infarction was detected via cranial MRI. We postulated that the underlying cause was the contrast agent’s high viscosity, which impeded blood flow in the cerebral arteries and resulted in neurotoxicity.

Additionally, CIE can manifest differently in individuals, and there have been cases in which patients developed CIE despite receiving a minute amount of contrast agent or undergoing intravenous contrast-enhanced CT scans.^[10–13]^ In addition to memory loss, dysarthria, cortical blindness, fever, dizziness, and headache, CIE can also present with a range of clinical manifestations. These may include gastrointestinal symptoms, such as nausea, vomiting, and abdominal distention; psychiatric symptoms, such as confusion, delirium, and loss of consciousness; and neurological impairments, such as limb movement disorders, optic nerve and motoneural dysfunction, memory loss, and even cardiopulmonary arrest.^[13–15]^

Clinical professionals may encounter challenges in diagnosing or differentiating certain symptoms of CIE, as they can be easily mistaken for those of primary cerebrovascular lesions. Brain ischemia or cerebral hemorrhage are 2 potential imaging findings in CIE patients. To distinguish between cerebral ischemia and CIE, it has been suggested that the apparent diffusion coefficient (ADC) be employed. ADC measures the signal intensity of diffusion-weighted imaging images in cranial MRI. In patients with CIE, there is no abnormal intensity, while in patients with acute ischemic stroke and cytotoxic edema, a decrease in water diffusion in the infarcted tissue results in a decrease in ADC.^[16]^ Indeed, there have been instances of CIE where the symptoms and imaging findings do not correlate or where there are no imaging characteristics present at all.^[17,18]^

The patient in question developed the aforementioned symptoms following DCB treatment, and CIE was diagnosed through a combination of these symptoms and diffusion-weighted imaging images from a cranial MRI. The main approach to treating CIE is symptomatic, which involves adequate hydration to aid in the discharge of the contrast agent, the use of glucocorticoids to reduce neurotoxic effects, with dexamethasone being the most commonly used medication, maintaining water-electrolyte balance, providing anti-epileptic medications in the case of epilepsy, and administering calcium antagonists if vasospasm occurs. After treatment, this patient experienced a rapid remission of numerous symptoms.

In our view, the purpose of this case report is to raise awareness about the variable prognosis of CIE. It should be noted that not all CIE symptoms improve rapidly with treatment, particularly those related to nervous system damage, including visual disturbances, diplopia, and oculomotor dysfunction, which may be caused by optic and oculomotor nerve injury. Although most patients with CIE have a self-limited course and recover within 72 hours without sequelae, Qiu et al^[19]^ reported a patient with CIE who required 19 days to recover after undergoing cerebral angiography. The case report by Leong et al^[20]^ reported a patient who developed CIE after undergoing intracranial aneurysm coiling, and 1 year later, the patient still suffered from chronic left-sided limb spasticity and was diagnosed with irreversible CIE. Among the CIE cases they analyzed, the median duration for neurological function recovery was 2.5 days. Despite receiving prompt therapy, the patient’s ocular problems took a slow and gradual recovery course and were ultimately resolved after a period of 3 months.

In addition to attending to the physical manifestations of the disease, it is crucial to pay close attention to the psychological well-being of patients. Psychological distress and despondency can exacerbate symptoms and delay recovery. During follow-up, we observed that the patient’s psychological distress post-CIE onset was due to social comparison, as she had acquaintances who underwent similar coronary procedures without encountering CIE, which made her fear that she would never fully recover. An all-encompassing approach to the management of CIE should address both the physical and psychological aspects of the disease to ensure optimal outcomes for patients.

## 4. Conclusion

CIE manifests in a plethora of ways, thereby rendering diagnosis and differentiation arduous. Symptomatic convalescence may be prolonged, and CIE can manifest in coronary DCB procedures, as ascertained from this patient’s case. Clinicians, in addition to expeditiously identifying and treating CIE, must provide patients with psychotherapeutic intervention to buttress their psychological welfare, which fosters their recuperation.

## Author contributions

**Conceptualization:** Lixin Liu, Xueying Chen.

**Data curation:** Kaiyuan Kong, Anyong Chen, Guoliang Yang, Ronghua Gao, Shaohui Zhang.

**Formal analysis:** Kaiyuan Kong, Anyong Chen, Guoliang Yang, Ronghua Gao, Shaohui Zhang, Lixin Liu.

**Funding acquisition:** Xueying Chen.

**Investigation:** Kaiyuan Kong, Anyong Chen, Guoliang Yang, Ronghua Gao, Shaohui Zhang, Lixin Liu, Xueying Chen.

**Project administration:** Ronghua Gao, Shaohui Zhang, Lixin Liu, Xueying Chen.

**Writing – original draft:** Kaiyuan Kong.

**Writing – review & editing:** Anyong Chen, Guoliang Yang, Ronghua Gao, Shaohui Zhang, Lixin Liu, Xueying Chen.
